# Risks, Benefits, and the Optimal Time to Resume Deep Vein Thrombosis Prophylaxis in Patients with Intracranial Hemorrhage

**DOI:** 10.7759/cureus.5827

**Published:** 2019-10-02

**Authors:** Saman Farr, Harjyot Toor, Tye Patchana, Stacey Podkovik, James G Wiginton, Raed Sweiss, Margaret Rose Wacker, Dan E Miulli

**Affiliations:** 1 Neurosurgery, Riverside University Health System Medical Center, Riverside, USA; 2 Neurosurgery, Riverside University Health System Medical Center, Moreno Valley, USA; 3 Neurosurgery, Arrowhead Regional Medical Center, Colton, USA

**Keywords:** intracranial hemorrhage, deep vein thrombosis, dvt prophylaxis

## Abstract

Introduction

It is common to start all patients on chemical prophylaxis for deep vein thrombosis (DVT) in order to decrease the risk of venous thromboembolism (VTE) and the associated adverse effects, including the potential for fatal pulmonary embolism (PE). There is no consensus in the literature on the optimal time to resume chemical DVT prophylaxis in patients who present with intracranial hemorrhage requiring neurosurgical intervention. The practice is variable and practitioner dependent. There can be difficulty in balancing the increased risk of further intracranial hemorrhage versus the benefit of starting DVT prophylaxis to prevent VTE.

Method

A retrospective review of patients that had diagnosis of intracranial hemorrhage (ICH) defined as epidural hematoma (EDH), subdural hematoma (SDH), or intra-parenchymal hematoma (IPH), was performed using the neurosurgical census at our institution. The review consisted of adult patients greater than 18 years old with a diagnosis of intracranial hemorrhage. Type of intracranial hemorrhage, method of neurosurgical intervention (whether surgical, bedside procedure, or both), day post-procedure prophylaxis was resumed, and the type of chemical prophylaxis used (subcutaneous heparin (SQH) versus enoxaparin) were recorded. The patient’s sex, Glasgow Coma Scale on presentation and discharge, length of hospital stay, and length of intensive care unit (ICU) stay were also recorded. Patients with previously diagnosed bleeding dyscrasia, previously diagnosed DVT or PE, patients without post-procedure cranial imaging (CT or MRI), and patients without post-procedure duplex ultrasound for DVT screening were excluded. Patients were monitored with head CT for possible expansion of ICH after resumption of therapy. Furthermore, we investigated whether the patient developed an adverse effect such as venous thromboembolism including deep vein thrombosis and/or pulmonary embolism during the post-procedure period when they were not on chemical prophylaxis.

Results

A total of 94 patients were analyzed in our study. Nine (9.6%) had an EDH, seventeen (18.1%) had an IPH, and sixty-eight (72.3%) had a SDH. The three most common procedures were craniectomy (28.7%), craniotomy (34%), and subdural drain placement (28.7%). The most common agent for chemical DVT prophylaxis was SQH in 78% of patients. There was no statistically significant association between type of chemical DVT prophylaxis used with respect to either ICU length of stay or hospital length of stay. Change in GCS (the difference of GCS on presentation versus on discharge) was found to have statistically significant relationship with the use of chemical DVT prophylaxis. Furthermore, patients were found to have no statistically significant association with re-bleed or new hemorrhage upon starting chemical DVT prophylaxis, regardless of the type of ICH.

Conclusion

The rates of DVT diagnosis did not seem to be significantly affected by the specific type of chemical prophylaxis that was used. ICU and hospital length of stay were not adversely affected by starting prophylaxis for VTE in patients with ICH. On the contrary, an improvement in GCS (on presentation versus discharge) was associated with starting chemical DVT prophylaxis in ICH patients within 24 hours post-procedure.

## Introduction

Deep vein thrombosis (DVT) and pulmonary embolism (PE) are a subset of venous thromboembolism (VTE). VTE has classically been summarized using “Virchow’s Triad” paradigm which includes endothelial damage to the vessel wall, a hypercoagulable state that promotes a coagulation event, and hemodynamic changes which cause a shift or disturbance in the blood flow pattern. Although Virchow described his triad in 1856, DVT and VTE continues to be a major preventable cause of morbidity and mortality worldwide.

VTE in general is considered to be a condition affecting older populations - as age increases, the rate of PE and DVT also increases. Incidence prior to late adolescence is rare [1]. During childbearing years (16 to 44 years), women are reported to have a higher incidence compared to their male counterparts. In individuals above the age of 45, men have a higher rate of incidence [1]. Overall, men have been found to have a higher rate of 130 per 100,000 compared to 110 per 100,000 for women when rates are adjusted for age [2,3].

The estimated annual incidence of VTE among individuals of European descent ranges from 104 to 183 per 100,000 person-years [1]. These rates are grossly similar to those reported for stroke in the United Kingdom and United States [4,5]. African-Americans are noted to have a higher overall VTE incidence, while Asians, Asian Americans, and Native Americans have been reported as having a lower incidence [6-12].

The reported incidence rates for PE (with or without DVT) range from 29 to 78 per 100,000 person-years whereas the rates for DVT alone (without PE) range from 45 to 117 per 100,000 person-years. While the literature on overall trends in VTE, DVT, and PE are limited, reports indicate that the rate either remained constant or increased from 1981-2000, with a subsequent substantial increase in the incidence of PE between 2001 and 2009 [1]. Some sources suggest that the observed increase may in part have been due to increased use of CT pulmonary angiography in the diagnosis of PE [13].

The endothelium of blood vessels plays an important role in the prevention of VTE, and injury to this layer can occur during surgery or with venous catheter insertion in patients in the intensive care unit (ICU). This can reduce flow in the affected vessel as well as expose subendothelial tissue factor, both of which activate the clotting cascade potentially resulting in the formation of a thrombus, as described in Virchow's Triad.

VTE formation is of particular importance in the neurosurgical patient with rates varying between 1 and 34% [14-19]. Based on national data, the reported rates of symptomatic DVTs range between 1-4% [20-23]. This difference is likely explained by the increased rates of surveillance and subsequent detection in recent years. The incidence in neurosurgical patients that were actively screened with doppler ultrasound, despite having no associated symptoms and receiving at least one form of DVT prophylaxis, was found to be between 3 and 16% [24-26].

## Materials and methods

In this retrospective study, we reviewed data from patients at a Level 2 Trauma Center in southern California between 2016-2019. We queried the internal patient census from the Neurosurgery Department, looking at adult patients (greater than 18 years old) that had a diagnosis of intracranial hemorrhage (ICH). These patients were subsequently classified by the type of hemorrhage (epidural hematoma (EDH), subdural hematoma (SDH), intra-parenchymal hematoma (IPH), or subarachnoid hemorrhage (SAH)).

The patient charts were then reviewed for types of intracranial hemorrhage, type of neurosurgical intervention (whether the patient underwent surgical intervention, a bedside procedure, or both), day post-intervention chemical DVT prophylaxis was resumed, and the type of chemical prophylaxis used (subcutaneous heparin versus enoxaparin, etc.). Other variables that were studied included the patient’s sex as well as the Glasgow Coma Scale on presentation.

Patients with a previously diagnosed bleeding dyscrasia, previously diagnosed DVT or PE, patients without post-intervention cranial imaging (CT or MRI to evaluate for stability of intracranial hemorrhage), and patients without post-intervention Duplex Ultrasound for DVT screening were all excluded. We also analyzed whether the patient had an expansion of intracranial hemorrhage or development of a new hemorrhage after resumption of chemical DVT prophylaxis, as evaluated by CT or MRI imaging.

Furthermore, we investigated whether the patient developed an adverse event such as development of venous thromboembolism, including deep vein thrombosis and/or pulmonary embolism during the post-procedure period while they were not on any chemical prophylaxis. Outcome measures analyzed included length of ICU stay, length of hospital stay, and Glasgow Coma Scale on discharge.

## Results

After application of inclusion and exclusion criteria, 94 patients were identified and analyzed in our study. Of these, twenty-one (22.3%) were female, and seventy-three (77.7%) were male (Table 1). Age ranged from 18-87, with a mean of 53.39 and standard deviation of 20.50. Patients were stratified for hemorrhage type (Table 2). Nine patients (9.6%) presented with EDH, seventeen (18.1%) presented with IPH, and sixty-eight (72.3%) presented with SDH. Patients were further stratified by type of procedure performed (Table 3). The three most common procedures were craniectomy (28.7%), craniotomy (34%) and subdural drain placement (28.7%). Type of chemical prophylaxis used patients received was recorded (Table 4), the most common of which was SQH in (78% of patients, n=73). At our institution SQH is given as 5,000 units administered subcutaneously every 8 hours.

**Table 1:** Patient demographic breakdown with respect to sex.

	Frequency	Percent
Female	21	22.3%
Male	73	77.7%
Total	94	100%

 

**Table 2:** Type of hemorrhage: this table describes the breakdown with respect to type of intracranial hemorrhage. EDH: epidural hematoma, IPH: intraparenchymal hematoma, SDH: subdural hematoma.

	Frequency	Percent
EDH	9	9.6%
IPH	17	18.1%
SDH	68	72.3%
Total	94	100%

**Table 3:** Type of procedure: this table shows the breakdown of patients based on the type of procedure performed. (SDD: subdural drain, VPS: ventriculoperitoneal shunt, IPH drain: intraparenchymal hemorrhage drain)

	Frequency	Percent
Craniectomy	27	28.7%
Cranioplasty	1	1.1%
Craniotomy	32	34.0%
IPH drain	3	3.2%
SDD	27	28.7%
VPS	2	2.1%
Other	2	2.1%

**Table 4:** Type of chemical DVT prophylaxis: patients were analyzed based on the type of chemical prophylaxis that was administered. (SQH: subcutaneous heparin)

	Frequency	Percent
None	8	8.5%
SQH	79	84.0%
Enoxaparin	2	2.1%
SQH/Enoxaparin	5	5.3%
Total	94	100.0%

Length of stay (LOS) (both in the ICU and hospital), as well as GCS (on presentation and discharge) were analyzed (Table 5). No statistically significant association between type of chemical DVT prophylaxis used with respect to ICU length of stay (p-value = 0.627) or hospital length of stay (p-value = 0.399) was found.

**Table 5:** Outcome measures: LOS (length of stay) in the ICU (intensive care unit), hospital as well as GCS (Glasgow coma scale) both on presentation and on discharge.

	Minimum	Maximum	Mean	STD
LOS in ICU	0	49	11.1	9.73
LOS in hospital	2	74	15	12.84
GCS on presentation	3	15	10.89	4.38
GCS on discharge	3	15	11.63	4.41

None of the 9 patients with EDH had expansion of hemorrhage or new hemorrhage upon starting chemical DVT prophylaxis as evidenced by repeat CTH. Incidence of rebleed in patients with SDH was six but there was found to be no statistically significant association with rebleed or new hemorrhage upon starting chemical DVT prophylaxis (p-value = 0.527). Although one patient with intraparenchymal hemorrhage experienced rebleeding, this patient had not been placed on chemical DVT prophylaxis. Therefore, there was no statistically significant association with new hemorrhage or expansion of hemorrhage (p-value = 0.052). Rate of DVT diagnosis and the type of chemical DVT prophylaxis also had no statistical significance (p-value = 0.872). A total of two patients in the cohort had a diagnosis of DVT.

## Discussion

In the face of hemorrhage expansion following prophylaxis for DVTs, when, how much to use, and what agent is best is a controversial topic in neurosurgical care. This patient population is especially prone to venous thromboembolism secondary to increased age, ventilator support, venous injury, and major surgeries [27]. Patients with intracerebral hemorrhage additionally often present following polytrauma, which further increases the risks of DVTs. Prior studies have found a 20% incidence of DVT in a small cohort of twenty patients with isolated head injury [28]. A further study assessing VTE using weekly duplex doppler studies of the lower extremities, demonstrated a 25% incidence of positive duplex scan [29]. Therefore, the risk of expansion of hemorrhage must be tempered against the risk of fatal pulmonary embolism secondary to venous thromboembolism. However, in our study, only 2.1% of patients were diagnosed with DVT.

At our institution, we begin DVT prophylaxis 24 hours after stability has been demonstrated on repeat imaging. A more precise timing protocol may be elucidated in the future with further studies. We did not find a statistically significant association between type of chemical DVT prophylaxis used with respect to ICU length of stay or hospital length of stay.

Although expansion of hemorrhage is a feared complication upon initiation of chemical DVT prophylaxis in ICH, there was no identified statistically significant association with new bleed or hemorrhage after starting prophylaxis. We also did not find a significant relationship between rate of DVT diagnosis and the type of chemical DVT prophylaxis. Though our patient population was small, we have demonstrated that our timeline of beginning DVT prophylaxis 24 hours after a stable scan did not lead to significant hemorrhage expansion or rebleed when compared across multiple hemorrhage types.

Our retrospective study was likely limited by the number of patients (n=94). Statistical significance may be reached in future studies with the addition of a larger patient cohort.

## Conclusions

At our institution, chemical DVT prophylaxis is initiated 24 hours after stable imaging of the intracranial hemorrhage has been documented. Based on the results of our retrospective study, several conclusions can be drawn. The first is that our neurosurgical patients did not have a statistically significant increase in expansion of hemorrhage or development of new hemorrhage after starting chemical DVT prophylaxis as assessed by repeat CTH after starting prophylaxis. This was true regardless of the type of intracranial hemorrhage that the patient was diagnosed with (EDH, SDH, or IPH), thus showing the safety of chemical DVT prophylaxis in these patients. The rates of DVT diagnosis did not seem to be significantly affected by the specific type of chemical prophylaxis that was used (subcutaneous heparin, enoxaparin, etc.). This is consistent with previous literature. Length of hospital or ICU stay was not adversely affected by the patient having been started on chemical DVT prophylaxis. Further prospective studies to better elucidate timing and type of DVT prophylaxis are warranted as well as the possibility of shorter ICU and hospital stays due to fewer DVT.
